# Moroccans’ Views on Resuscitation According to Presumed Degree of Disability: A Cross-Sectional Study

**DOI:** 10.7759/cureus.33460

**Published:** 2023-01-06

**Authors:** Sabah Benhamza, Laila Lahlou, Mohammad Khalayla, Mohamed Lazraq, Youssef Miloudi, Abdelhak Bensaid, Najib El Harrar

**Affiliations:** 1 Anesthesiology and Critical Care, Ibn Rochd University Hospital of Casablanca, Hassan II University of Casablanca, Casablanca, MAR; 2 Epidemiology and Public Health, Faculty of Medicine and Pharmacy, Ibn Zohr University, Agadir, MAR

**Keywords:** morocco, decision-making process, palliative and supportive care, réanimation, handicap

## Abstract

Introduction: According to the World Health Organization (WHO), disability is a public health problem that can be difficult to manage medically and financially. Disability can either be innate or develop after resuscitation. Therefore, the decision regarding whether to resuscitate a patient or not raises certain ethical questions, especially in the context of a Muslim country such as Morocco.

Aim: The main aim of this study is to survey the public’s opinions regarding their willingness to be resuscitated or have their relatives be resuscitated based on their foreseeable degree of disability.

Methods: This cross-sectional study was conducted over a 10-month period and employed a self-administered questionnaire. The participants included were all adult (i.e., over 18 years of age) Moroccan nationals, and they were selected regardless of their religious identity. Moreover, the modified Rankin Scale (mRS) was used to measure the participants’ foreseeable degree of handicap. The participants were divided into two groups: healthcare workers and non-healthcare workers.

Results: In total, 1083 questionnaires were retained. The average age of the participants was 30 (± 8) years, with the male-to-female sex ratio being 0.78. Moreover, 39.6% of the participants were healthcare workers. It was found that compared to the non healthcare workers, the healthcare professionals were more willing to be resuscitated themselves and have resuscitation performed on their relatives, but only when the degree of foreseeable disability was estimated to be absent or insignificant, whereas they were less willing to be resuscitated and have resuscitation performed on their relatives when the degree of foreseeable disability was estimated to be mild or higher.

Conclusion: In conclusion, there should be a pre-established procedure, along with a legislative and multidisciplinary framework, within the hospital structures in order to help in the decision-making process regarding whether to resuscitate a patient or not.

## Introduction

The World Health Organization (WHO) estimates that 15% of the world’s population is exposed to some form of disability, and 190 million people over the age of 15 have significant functional difficulties that would require healthcare. Therefore, disability is considered a public health problem, as it can take many forms and lead to significant healthcare needs that are difficult to address in low-income countries. Moreover, according to WHO, more than half of the people with disabilities cannot afford adequate medical care, which is higher than in the case of people without disabilities [1].

The core mission of the intensive care unit (ICU) is to take care of patients with life-threatening acute organ failure, which is often caused by multiple factors. However, although the care received in an ICU is often life-saving, many survivors end up with new or worsening functional, cognitive, or mental disabilities that may persist over time [2,3].

In theory, resuscitation should be performed in all cases of organ failure, including circulatory arrest, but some questions remain: should resuscitation be performed in cases where the chances of survival are estimated to be very low (such as in case of certain cancer pathologies where the treatment has failed), and is this resuscitation a wish of the patient or only of the attending physician? In Morocco, a Muslim country where social traditions play an important role in decision-making, the choice to resuscitate a patient is generally at the discretion of the attending physician.

Our study aims to survey the public opinions regarding the concept of resuscitation based on the presumed degree of foreseeable disability (whether their own or that of their relatives) in order to determine whether, in the Moroccan context, aggressive resuscitation could be the result of the will of the patients, their relatives, or only doctors.

## Materials and methods

We conducted a cross-sectional study over a period of 10 months, from June 2019 to February 2020, through a questionnaire concerning the entire Moroccan population. The population was divided into two groups: the first representing healthcare professionals, including doctors and nurses, and the other representing non-healthcare workers. The participants included adults (≥ 18 years old) of Moroccan nationality who were selected regardless of their religious background, while Moroccans residing abroad as well as anyone who did not complete the questionnaire were excluded from the study.

The data was collected through a self-administered questionnaire, which was delivered either by hand or in electronic format to the two groups of participants. Prior to this, the questionnaire was tested on a few doctors as well as non-healthcare professionals and validated after modifying the errors, reformulating the questions, and calculating the average response time, which was estimated to be four minutes.

The questionnaire was structured in two parts. The first part concerned the socio-demographic and professional characteristics of the participants, along with their medical-surgical, toxic, and neoplastic pathological history with the different stages of cancer: stage 1 (single and small tumour), stage 2 (larger tumour), stage 3 (the tumour invades the lymph nodes or surrounding tissues), and stage 4 (the presence of metastases in other organs distant from the initial tumour). Meanwhile, the second part focused on the overall assessment of the benefit of performing resuscitation according to their presumed degree of disability.

For the reference test, we opted for the modified Rankin Scale (mRS), which reveals a score for a patient’s overall autonomy. The scale has five levels [4]: 0 for the absence of symptoms, 1 for the absence of disability but with symptoms present (i.e., the patient is able to perform all usual activities), 2 for mild disability (i.e., the patient is unable to perform all of the above activities but able to do some without assistance), 3 for disability moderate (i.e., the patient needs assistance but is able to walk independently with or without a cane), 4 for moderately severe disability (i.e., the patient is unable to walk unaided and unable to manage bodily needs without assistance), and 5 for severe disability (i.e., the patient is bedridden, incontinent, and requires constant supervision and nursing care). Additionally, we chose this scale for our study because of the simplicity of its items, which we expected would be more easily understood by the general Moroccan population. In our study, this scale was introduced with the intention to compare the variations in the opinions of the participants regarding resuscitation according to the degree of foreseeable handicap. The items were detailed in the questionnaire in order to explain the expected degree of disability.

The statistical analysis was performed using SPSS 13.0 (SPSS Inc., Chicago, IL, USA) software. This data analysis was carried out in two phases: a descriptive phase and an analytical phase. In the description phase, the results were represented as numbers and percentages for the categorical variables, and the measures of central tendency and dispersion were used for the quantitative variables, while in the analytical phase, we studied the association between the participants’ sociodemographic factors and their opinions regarding resuscitation based on the presumed degree of physical disability (mRS stages). The comparison of the percentages was carried out by using the Chi-squared test for the categorical variables in case the theoretical numbers were greater than 5. Moreover, the comparison of the two groups of responders was carried out by using the Student’s t test for the quantitative variables. Furthermore, the association between the subgroups of each variable was studied by using binary logistic regression in univariate analysis, where the odds ratios (OR) with confidence intervals (CI) at 95% were presented, and a p-value < 0.05 was considered statistically significant.

The questionnaire was published and sent to participants with a description explaining the purpose of our study. Moreover, they were informed that their participation was voluntary and anonymous and that they were free to refuse participation in the study.

## Results

We distributed 1500 questionnaires, out of which 1364 (90.9%) were collected, 1083 (79.4%) retained, and 281 (20.6%) rejected. The average age of the participants was 30 (± 8) years, with the male-to-female sex ratio being 0.78. The 26-40 age bracket accounted for 52.1% of all the participants, while 1.7% of them were over 55 years old. Moreover, 87.8% of the participants were of middle socio-economic level, while 2.9% were of low socio-economic level. As regards education, 96% of the participants had received higher education, 3.20% had received secondary education, and the rest had either received primary education or never been to school. Furthermore, healthcare workers constituted 39.6% of our participants, of which 23.5% were general practitioners, 8.4% were specialists, and 7.7% were nurses. Among the medical specialists, 48.3% were anaesthesiologists/resuscitators. As regards the participants who were not healthcare workers, 12% were unemployed, 52% were senior managers, and 19% were students. Additionally, 0.8% of the participants had cancer, out of which 44.4% were at stage 2 and the rest at stage 4. Besides, 2.7% had systemic diseases, 3% were hypertensive, 3.4% were diabetic, and 7% were asthmatics.

We found that 88.6% of the participants wanted their relatives to be informed about all the details about their condition if they were to be admitted in an ICU, while 98.6% wanted to know everything about their relatives’ condition, including the severity, if they were to be admitted in an ICU. Out of all the participants for whom the degree of foreseeable disability was estimated at an mRS score of 1, 63.5% wanted to be resuscitated and 58.9% wanted their relatives to be resuscitated, whereas out of the ones for whom the degree of foreseeable disability was estimated at an mRS score of 5, 20.5% wanted to be resuscitated and 27.3% wanted their relatives to be resuscitated. As regards the healthcare workers, out of the participants for whom the degree of foreseeable disability was estimated at an mRS score of 5, none wanted to be resuscitated and 99.6% also did not want their relatives to be resuscitated. Moreover, for the same degree of foreseeable disability, 66.1% of the general population did not want to be resuscitated and 55.7% did not want their relatives to be resuscitated.

This study found that there is a statistically significant relationship between the different age groups and the decision to resuscitate according to the stages of mRS for oneself or for a relative (Table 1). 

**Table 1:** Willingness health workers for oneself and for a relative according to the degree of disability and according to the age of the participants mRS: modified Rankin Scale

		For one self		For relative	
mRs	Age groups (years)	n(%)	P	n (%)	P
mRs 0	15-25	350 (86.6%)	<0.0001	297 (37.6%)	NS
	26-40	366 (64.9%)	<0.0001	423 (53.5%)	NS
	41-55	62 (63.9%)	NS	62 (7.8%)	0.036
	Over 55	18 (100.0%)	0.010	8 (1%)	0.006
mRs 1	15-25	318 (78.7%)	<0.0001	318 (78.7%)	<0.0001
	26-40	370 (65.6%)	NS	288 (45.14%)	<0.0001
	41-55	0 (0.0%)	<0.0001	53 (8.3%)	NS
	Over 55	0 (0.0%)	<0.0001	0 (0.0%)	<0.0001
mRs 2	15-25	295 (73.0%)	<0.0001	128 (24.2%)	<0.0001
	26-40	189 (33.5%)	<0.0001	287 (54.2%)	NS
	41-55	97 (100.0%)	<0.0001	97 (18.3%)	<0.0001
	Over 55	18 (100.0%)	<0.0001	18 (3,.4%)	<0.0001
mRs 3	15- 25	54 (13.4%)	<0.0001	107 (29.9%)	<0.0001
	26-40	201 (35,6%)	0.018	206 (57.5%)	0.011
	41-55	78 (80.4%)	P <0.0001	35 (9.8%)	NS
	Over 55	18 (100.0%)	P <0.0001	10 (2.8%)	0.041
mRs 4	15- 25	54 (13.4%)	P <0.0001	106 (44.4%)	0.011
	26-40	139 (24.6%)	P <0.0001	88 (36.8%)	0.027
	41- 55	35 (36.1%)	P <0.0001	35 (14.6%)	<0.0001
	Over 55	10 (55.6%)	0.001	10 (4.2%)	0.001
mRs 5	15- 25	54 (13.4%)	P <0.0001	60 (20.3%)	<0.0001
	26-40	123 (21.8%)	NS	188 (63.5%)	P <0.0001
	41-55	35 (36.1%)	P <0.0001	35 (36.1%)	P <0.0001
	Over 55	10 (55.6%)	P <0.0001	35 (11.8%)	NS

We observed that women were more willing to be resuscitated than men if the predicted degree of disability was equal to an mRS score of 1 (OR = 1.529; 95% CI = 1.188-1.969; p = 0.001) and less willing to be resuscitated than men when the predicted degree of disability was equal to an mRS score of 5 (OR = 0.612; 95% CI = 0.450-0.832; p = 0.002). Moreover, women were less likely than men to want their relatives to be resuscitated when the predicted degree of disability was equal to an mRS score of 2 (OR = 0.604; 95% CI = 0.474-0.769; p < 0.0001), mRS score of 3 (OR = 0.510; 95% IC = 0.392-0.664; p < 0.0001), or mRS score of 4 (OR = 0.528; 95% IC = 0.390-0.714; p < 0.0001). Furthermore, a university level of education was a risk factor for refusing resuscitation for oneself when the predicted degree of disability was strictly greater than an mRS score of 1 (Table 2).

**Table 2:** desire for resuscitation for oneself and for a loved one according to the degree of disability and the level of education of the participants. mRS: modified Rankin Scale

		For one self			For a relative		
mRs	Level of education	P	OR	IC95%	P	OR	IC95%
mRs 0	University	<0.0001	42.807	13.129 -139.577	<0.0001	6.818	3.504 – 13.266
	others						
mRs 1	University	<0.0001	4.798	2.434 – 9.455	<0.0001	5.782	2.744 – 12.180
	others						
mRs 2	University	<0.0001	0.466	0.237 – 0.917	0.005	0.401	0.207 – 0.778
	others						
mRs 3	University	<0.0001	0.116	0.055 – 0.245	<0.0001	0.200	0.103 – 0.388
	others						
mRs 4	University	<0.0001	0.074	0.036 – 0.153	<0.0001	0.109	0.056– 0.213
	others						
mRs 5	University	<0.0001	0.098	0.050 – 0.192	<0.0001	0.149	0.077 – 0.290
	others						

Regardless of the predicted degree of disability, the highest percentages of participants seeking resuscitation for themselves or their relatives were those belonging to the middle socioeconomic group, followed by those in the high socioeconomic group (Table 3). 

**Table 3:** Willingness for resuscitation for oneself and for a relative according to the degree of disability and the socio-economic level of the participants. mRS: modified Rankin Scale

		For one sel		For a relative	
mRs	socio-economic level	N (%)	P	N (%)	P
mRs 0	High	63 (7.9%)	0.008	69 (8.7%)	NS
	Low	5 (0.6%)	<0.0001	5 (0.6%)	<0.0001
	Middle	728 (91.5%)	<0.0001	716 (90.6%)	<0.0001
mRs 1	High	64 (9.3%)	NS	51 (8.02%)	NS
	Low	5 (0.7%)	<0.0001	5 (0.8%)	<0.0001
	Middle	619 (90.02%)	0.004	582 (91.2%)	<0.0001
mRs 2	High	32 (5.3%)	<0.0001	31 (5.8%)	<0.0001
	Low	26 (4.3%)	0.001	26 (4.9%)	<0.0001
	Middle	541 (90.3%)	0.005	473 (89.2%)	NS
mRs 3	High	44 (12.5%)	NS	31 (8.7%)	NS
	Low	26 (7.4%)	<0.0001	26 (7.3%)	<0.0001
	Middle	281 (80.1%)	<0.0001	301 (84.12%)	0.008
mRs 4	High	38 (16%)	<0.0001	31 (13%)	0.028
	Low	26 (10.9%)	<0.0001	26 (10.9%)	<0.0001
	Middle	174 (73.1%)	<0.0001	182 (76.2%)	<0.0001
mRs 5	High	31 (14%)	0.008	31 (10.5%)	NS
	Low	26 (11.7%)	<0.0001	26 (8.8%)	<0.0001
	Middle	165 (74.3%)	<0.0001	239 (80.7%)	<0.0001

Additionally, healthcare workers were more interested in seeking resuscitation for themselves and relatives than the general population only at predicted degrees of disability equivalent to an mRS score ≤ 1 (Table 4).

**Table 4:** Willingness for resuscitation for oneself and for a  relative according to the degree of disability in the two groups of participants mRS: modified Rankin Scale

		For one self			For a relative		
mRs stages	Profession	P	OR	IC95%	P	OR	IC95%
mRs 0	non healthcare workers	<0.0001	30.786	16.135 - 58.739	<0.0001	1.847	1.385 - 2.464
	healthcare workers						
mRs 1	non healthcare workers	<0.0001	103.489	45.544 - 235.157	<0.0001	1.774	1.377 - 2.286
	Health workers						
mRs 2	non healthcare workers	<0.0001	0.012	0.008 - 0.019	<0.0001	0.401	0.312 - 0.515
	Health workers						
mRs 3	non healthcare workers	<0.0001	0.029	0.016 - 0.052	<0.0001	0.691	0.531 - 0.900
	Health workers						
mRs 4	non healthcare workers	<0.0001	0.030	0.014 - 0.065	<0.0001	0.344	0.246 -0.482
	Health workers						
mRs 5	non healthcare workers	<0.0001	0.502	0.469 - 0.536	<0.0001	0.018	0.008 - 0.040
	Health workers						

## Discussion

Based on the results of our study, it would appear that the desire to be resuscitated or have a relative be resuscitated decreases as the predicted degree of disability increases. This is relatively consistent with the results of many other studies. For instance, in a study conducted in Israel, Carmel [5] demonstrated that only 25% of the participants wanted to undergo a cardiopulmonary resuscitation in the event of irreversible physical disability while bedridden or in the event of incontinence, while a study conducted in the United States [6] found that 74.4% of the participants did not want to undergo treatment if the result did not spare serious functional disability. Among the studies analysing the opinion of doctors in the United States, one showed that more than 85% of doctors did not want to receive cardiopulmonary resuscitation in the event of brain injury [7]. Moreover, in a German survey conducted on the opinion of the general population regarding decompressive craniectomy for malignant hemispheric infarction [8], only a few participants were in favour of this potentially life-saving procedure if survival was associated with severe or moderately severe disability. This indicates that people are more concerned with the quality of their life than with the concept of living, as demonstrated by the ETHICATT study [9].

Additionally, increasing disability is generally associated with decreased quality of life [10,11], but there are many exceptions, with some researchers reporting good quality of life despite severe disability [12,13]. Some patients reported being happier and more satisfied with their quality of life than healthy people expected under the same circumstances. This phenomenon has been called "the disability paradox" and is partly explained by the ability of patients with a chronic illness or a disability to adapt to their situation [14,15]. In other words, reliance can become acceptable when the alternative is death. This brings us back to the question of the decision regarding whether to resuscitate patients or not as well as who should be able to estimate whether the foreseeable handicap of the patient is acceptable or not. Informing the patient and their family about the disease, the possible treatments, and the prognosis is an ethical obligation, especially in the case of intensive care patients who are generally unable to decide for themselves. Hence, the family must receive full information, unless the patient has previously objected to it.

In Morocco, the code of ethics makes it possible to inform the family when the prognosis is critical or fatal. Article 31 of the Moroccan Code of Ethics [16] stipulates: “A critical prognosis can legitimately be hidden from the patient. The fatal prognosis must only be revealed to him with the greatest circumspection: but it must generally be revealed to the family. The patient may prohibit this disclosure or designate the third parties to whom it must be made.”

In our study, we found that a majority of the participants in both groups wanted to know everything about their relatives’ state of health. Moreover, they also wanted their families to know everything about their condition, which is consistent with the results of a study on patients receiving palliative care for cancer, which found that most of these patients wanted their families to know everything about their disease [17]. This demonstrates that the family can play an important role in the care-giving process of intensive care patients. Consequently, in the case of a patient admitted to an ICU, the families and doctors work together to offer the patient the care that best meets their expectations (i.e., preferences and values). Thus, relatives can play a decision-making role (i.e., being attorneys for the patient) or an advisory role in these situations [18].

In this study, we found that as age increased (i.e., going over the age of 55), the participants became more reluctant about being resuscitated or having their relatives be resuscitated. In the other age groups, there was a disparity in the decisions that the participants made for themselves and the ones that they made for a relative; although the participants refused to be resuscitated if the predictable degree of disability was considered high, they still wanted their relatives to be resuscitated under the same conditions. This can be explained by the cultural context of the Moroccan Arab-Muslim population, where family ties are important and make it difficult to decide not to resuscitate a relative.

Moreover, in the Moroccan Arab-Muslim cultural context, the pyramidal family model with the patriarch at the top [19] is predominant, especially in households where the level of education is low. In such households, young women - who may be wives, sisters, or daughters - are culturally obligated to care for patients from their family with disabilities. This spirit of duty and solidarity could explain the desire to resuscitate a parent regardless of the degree of their disability. Furthermore, a high level of education could be considered responsible for the evolution of the Moroccan family pattern towards a nuclear family rather than a community-oriented family [19], especially in urban areas, which could explain why in such households death is more acceptable for oneself or for a relative if the alternative is a major disability that could become a burden on the family.

This study also elucidates a difference in the decision-making process between healthcare workers and the general population, undoubtedly because disability is a clearer concept in the minds of healthcare workers and, therefore, they are probably more willing to accept it over death as an alternative. The main limitation of this study is that the participants reacted to hypothetical situations and that their attitudes could change in real-life situations.

## Conclusions

This study provided information regarding the participants’ desire to be resuscitated or have their relatives be resuscitated. Based on the results, we suggest scheduling informational sessions for the general public in relation to the concept of disability through broadcasts on local television channels and in the form of testimonials and feedback in order to relate the experiences of patients with post-resuscitation disability and their families. We also propose that there be a pre-established procedure with a legislative and multidisciplinary staff within our hospital structures to help in deciding whether to resuscitate or not.
